# Graphical classification of DNA sequences of HLA alleles by deep learning

**DOI:** 10.1007/s13577-017-0194-6

**Published:** 2018-01-11

**Authors:** Jun Miyake, Yuhei Kaneshita, Satoshi Asatani, Seiichi Tagawa, Hirohiko Niioka, Takashi Hirano

**Affiliations:** 1 Graduate School of Engineering Science, Osaka University, 1-3 Machikane-Yama, Toyonaka, Osaka 560-8531 Japan; 2 Global Center for Medical Engineering and Informatics, Osaka University, Building A-301, 1-3 Yamadaoka, Suita, Osaka 565-0871 Japan; 3 Okinawa Institute of Advanced Sciences, Uruma, 904-2234 Japan; 4 Present Address: Okinawa Research Institute of Sentan Pharma Co. Ltd., Uruma, Okinawa 904-2234 Japan

**Keywords:** HLA, Allele, Artificial intelligence, Deep learning, Autoencoder

## Abstract

Alleles of human leukocyte antigen (HLA)-A DNAs are classified and expressed graphically by using artificial intelligence “Deep Learning (Stacked autoencoder)”. Nucleotide sequence data corresponding to the length of 822 bp, collected from the Immuno Polymorphism Database, were compressed to 2-dimensional representation and were plotted. Profiles of the two-dimensional plots indicate that the alleles can be classified as clusters are formed. The two-dimensional plot of HLA-A DNAs gives a clear outlook for characterizing the various alleles.

**Electronic supplementary material:**

The online version of this article (10.1007/s13577-017-0194-6) contains supplementary material, which is available to authorized users.

## Introduction

Deep learning, an artificial intelligence, has various potentials in the technologies as automatic driving, playing games, reading sentences, etc. In 2006, Geoffrey Hinton showed that multilayered neural networks is superior to principal component analysis (PCA) in classification performance [1]. Deep learning has extended its application range to speech recognition, general image recognition [2, 3], and estimating DNA splicing selectivity [4].

We have been aiming to develop a method to outlook the nature of genomic sequences. Because the genes are consisted by sequential combination of many nucleotides, usually several hundred to thousands, it is impossible to grip the structures, nature and meanings directly by our own intellectual ability. Some special parts of the genes are used as nameplates. However, such part [in many cases as SNPs (single nucleotide polymorphisms)] might not represent the whole structure of entire sequence. The difficulty came from the fact the sequence structure is too long (large in bps) beyond our intelligence nor analytical sciences to grip instantly. Deep learning is a method to project a complex system to another complex system, which human intelligence can recognize easier.

In this paper, we examined the sequences of DNA of human leukocyte antigen (HLA)-A. HLA is very important as it relates the function of immune reaction. Resistance against cancer growth, histocompatibility at transplantation of organs, etc., rely on HLA. It is not easy to grip the relation of the sequences, structures and functions in a simple way. A method to outlook such molecular sequences for understanding the characteristics is desired, which would be important in medical applications.

Grasping the complex system is usually difficult by analytical methods. Cognitional understanding (prospect, landscape, mapping, etc.) could be obtained by artificial intelligence “deep learning”. We studied how the characteristics of the gene could be expressed by using stacked autoencoder, which is one of the methods of “Deep Learning”. The spatial dispersion of HLA-A genes belonging to various alleles was examined by plotting them two-dimensionally. A clear graphical relationship of HLA-As was visible.

## Materials and methods

To acquire graphical relationships among the DNA sequences, we use deep learning technology. We have been examining the method specially for conceptual understanding of biological aspects as DNA sequences [5, 6].

HLA-A sequence data are obtained from The European Bioinformatics Institute (EMBL-EBI) (http://www.ebi.ac.uk/). The HLA-A dataset consists of 19 subtypes. The number of total sequences is 540 as shown in Table 1 (alleles with the data < 10 are avoided.).

**Table 1:** Data set of alleles: numbers of samples of alleles of HLA-A in the database

Allele	Number
A*01	30
A*02	164
A*03	28
A*11	60
A*23	12
A*24	96
A*26	41
A*29	15
A*30	20
A*31	30
A*32	14
A*33	30
Total	540

**Fig. 1:**
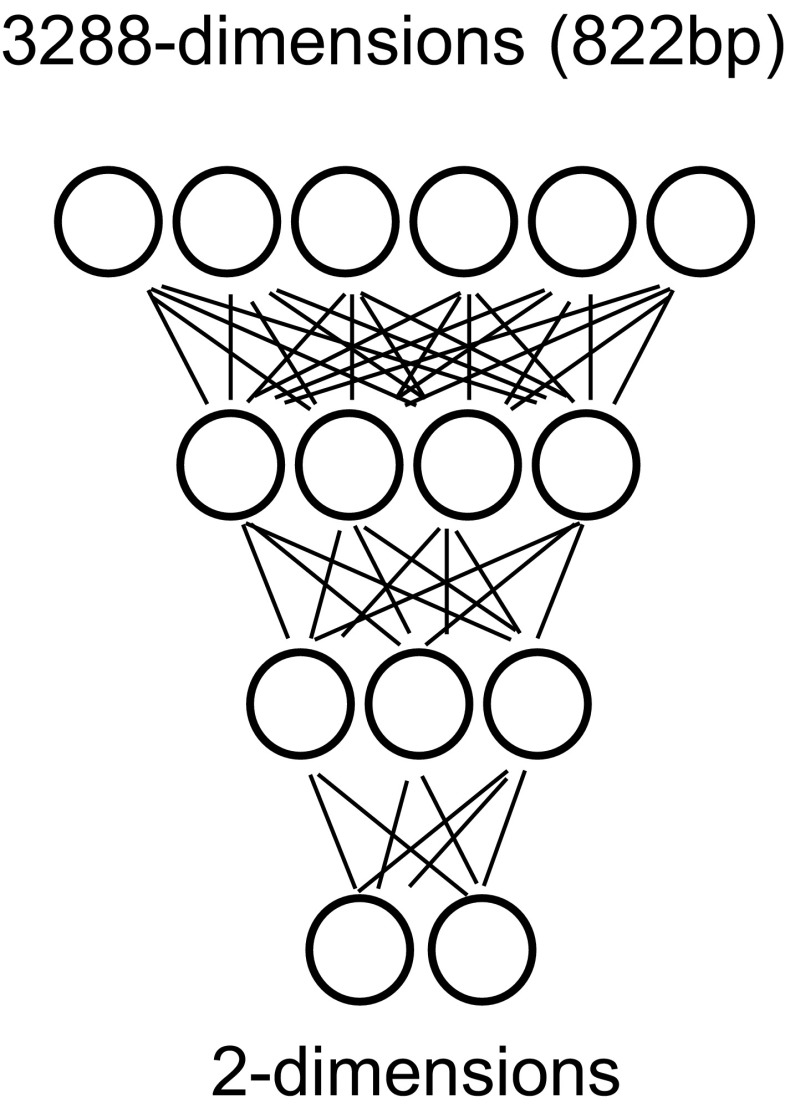
Schematic illustration of the compression process of autoencoder for HLA analysis. DNA sequence of HLA-A (822 bp) is regarded as the input layer. Resulted 2-dimensional layer is expressed as a dot on 2-D plane

Our method to abstract the 2-D feature of HLA-A DNA sequence consists of two procedures, (A) numerical conversion of DNA sequence and (B) its dimension reduction. (A) The numerical conversion is done by replacing DNA characters (A,G,C,T) into 4-D numerical vectors defined as shown in Table 2. (B) The dimension reduction is done by autoencoders. The autoencoder provides a nonlinear projection into the lower dimensional space by setting the smaller number of nodes in the middle layer in the autoencoder than that in the input (and output) layer. We reduce the dimension of numerically converted DNA sequences from 3288 (822 × 4) to 2, gradually (Fig. 1).

**Table 2:** Data set of alleles: attribution of bases to digits

Base	Attributed digit
A	1000
T	0100
C	0010
G	0001

In the method of stacked autoencoder, there are an *n*-dimensional input layer, an *n*′-dimensional intermediate layer and an n-dimensional output layer (*n* > *n*′). The network of the autoencoder is trained by input data as teacher data to reproduce input data at the output layer. It means that the lower dimensional data representation can maintain sufficient information and relationships of each input data in the lower dimensional feature spaces, if the trained autoencoder can reproduce the input data at the output layer. *n*′-Dimensional intermediate data are used to train the smaller autoencoder to create the more lower dimensional intermediate output. By repeating these processes, it is possible to obtain compressed two-dimensional data finally. As designed by Geoffrey Hinton [1], the information could be kept even after the compression, i.e., information of the DNA molecule of HLA-A could be interpreted even compressed to 2 dimensions.

We construct stacked autoencoder composed of five (in the case of numerical vector) autoencoders and train them through back-propagation and stochastic gradient descent to minimize the reconstruction error. Activation function is ReLU. Data with the dimension of 3288 are successfully compressed to 2 dimensional data with a certain accuracy. The layers have 3288, 1600, 800, 400, 150 and 2 nodes, respectively (input layer, four intermediate layers, and output layer). Consequently, the stacked autoencoder projects the input data to 2-dimensional feature representation. We use the deep learning library SIGMA-OU, which is an open source library developed and published by our group [7].

In addition to the binary numerical vector representation, we also use another data representation, document vector [8]. The method makes a histogram of words appearing in the sequence. The ‘word’ means an *l*-mer tiny sequence, given the tiny sequence length *l* (e.g. 4-mer words are ‘AAAA’, ‘AAAT’, ‘AAAC’, ‘AAAG’,…, ‘GGGC’, ‘GGGG’). In this research, we use its alternative overlapping mode under *l* = 5, and the number of all words is 1024. In experiments using this method, the histogram is 1024-dimensional vector of real numbers.

In case of document vector, the number of all words is 1024. A stacked autoencoder to be trained consists of four autoencoders. The intermediate layers of each autoencoder have 768, 384, 128 and 2 nodes, respectively. The number of the nodes and the layers is different from the case of using numerical vector. Various hyper parameters (e.g., the number of the layers, the number of the nodes, etc.) are examined and a set of nodes is empirically obtained which give a sufficiently low reconstruction error.

## Results and discussion

In this work, autoencoder of deep learning is applied as a method to extract the characteristics of long-chain DNA of HLA-A. The reconstruction errors in the compression process decreased effectively (see Supplemental Figs. 1 and 2) in the both cases of numerical vector and document vector. The two-dimensional expression might lack some information content of the gene characteristics as shown that the reconstruction error plateaued at approximately 0.2 > 0.0 in some stages of Supplement Figs. 1 and 2. However, the stacked autoencoder learns the characteristics as much as possible on the neural network and each stage maintained at least about 80% information of characteristic to be shown in the two-dimensional expression. Essential part of the information could be stored while being compressed.

**Fig. 2:**
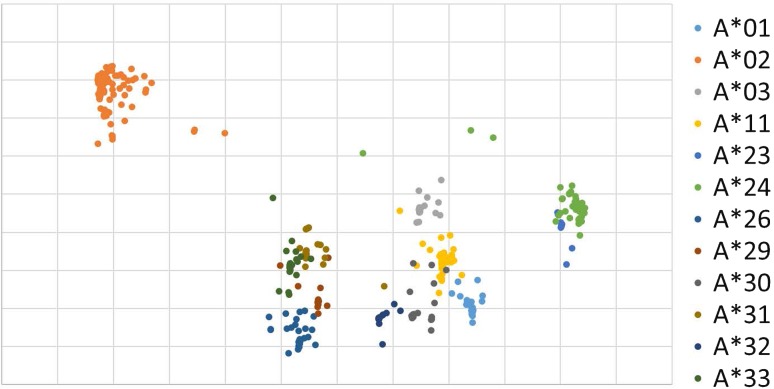
Graphical projection of HLA-A DNA onto 2-dimensional feature space. Colors of the dots corresponding the alleles, individually. Clusters of the dots are obviously related to the allele types expressed by different colors (A*01–A*33)

We compressed the DNA data from 3288 dimensions (822 bp) to 2 dimensions with using numerical vector (Fig. 2). Dots forming clusters are seen but each cluster does not represent one allele. Note the grid axis does not correspond to any physical meanings.

**Fig. 3:**
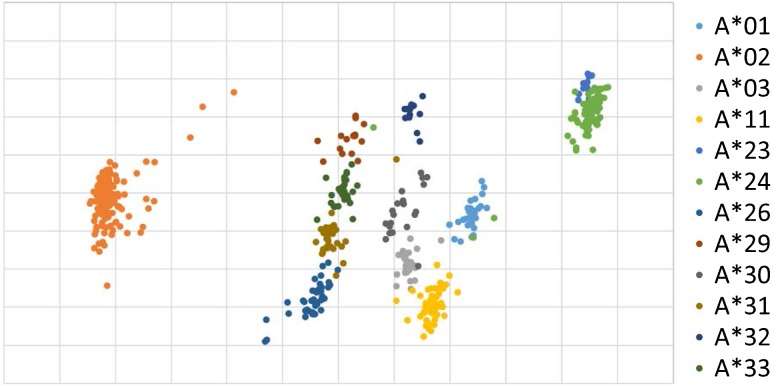
Histogram-based document vector analysis of HLA-A using autoencoder. Positions of alleles are different from Fig. 2 but each one looks much sharpened and independent from those of other alleles. The meanings of the distances and directions of alleles are under investigation but they could be correlated to the genetic differences for immune characteristics. Closer the positions should indicate the mutual similarities of the sequences

Aiming for the further improvement of feature extraction, the document vector is examined (Fig. 3). Histogram-based document vector is applied based on the study of [8]. It was used in our laboratory to overcome the differences of the length of DNA in various genes. Comparison between *n* = 4, 5, and 6 have been done and the result with *n* = 5 gave the most clear separations empirically. Also the cases of *n* > 6 were avoided to reduce the time of calculation.

Almost all the alleles are identified as own clusters. The separation provides a clear image of classification (Fig. 3). Formation of isolated clusters is dependent on the genes of the individual alleles. The mutual position of the clusters should be meaningful. In our study on mitochondrial DNA, the distances on the two-dimensional distribution described by autoencoder are related to the difference of DNA sequence and molecular clock [5]. Two-dimensional distribution of clusters could be the measure of their mutual relations but we need more examinations with biological data.

**Fig. 4:**
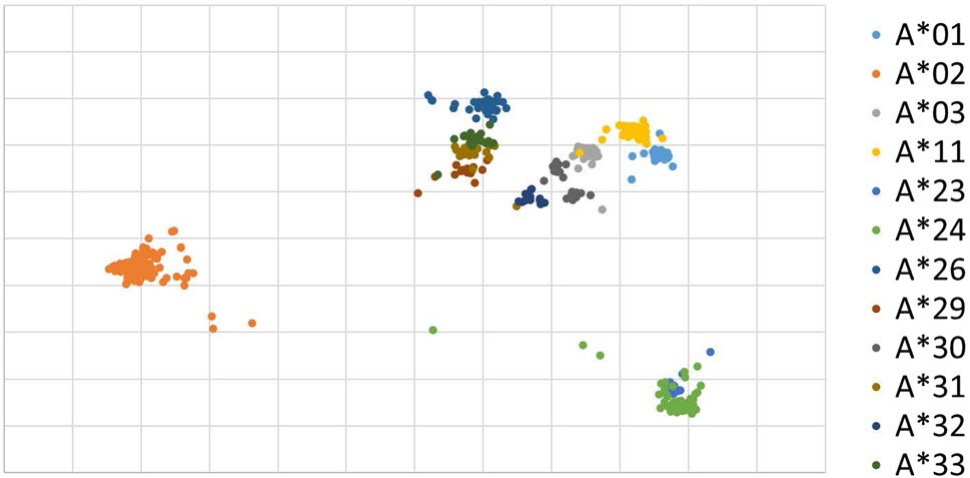
Histogram-based document vector analysis of HLA-A by PCA method. Positions of alleles are different from Fig. 3 and the clusters are less clearly organized and overlaps prevent simple identification of alleles

The same analysis was done using PCA method for comparison with using document vector (Fig. 4). Comparison of Figs. 3 and 4 indicates autoencoder analysis is superior to PCA methods in the cluster illustration. We assume at this moment, the clearer differentiations of clusters are derived from capability of autoencoder considering the sequences of the DNAs and repeated learning of them.

So far we experienced, number of samples, length of DNA base pars, and the nature of DNA affect the clustering. Usually the larger the number of training data gives the better resolution (separation of clusters). The longer the length of DNA sequence is the clearer in resolution in a certain extent. However, too much larger and/or longer the length DNA data make the calculation process complicated and time consuming. The best number and the length seem to differ by the nature of DNA and are under investigation. Grid scales are also affected by the conditions of calculation. For the ease of comparison, scales are set arbitrary (Figs. 2, 3).

Mutation causes various differences in the DNA of certain molecules. Comparison and classification of the sequences require measures. If the difference is limited, the number of differences should be the principal measure. But the larger scale and/or random mutations give a difficulty of preparing the measure. The degree of the difference/similarity of entire DNA sequences is hard to be defined mathematically in such complex system. We are studying autoencoder if it has a potential to give a conceptual view on the differences and functions.

The two-dimensional plane is a useful expression for intuitive grasping the characteristics of genes. Difference/relation of the alleles is simply overviewed. Distances between the clusters indicate the degree of differences of their sequences. Based on the analysis for human and relatives using autoencoder, we think the distance and the direction of the relation of the clusters indicate the molecular clock and genetical evolution pathway.

We should like to propose the autoencoder-based conceptual expression of the nature of HLA DNAs could give a tool for the research on the mechanism of immune system, giving solutions or indication of the medical analysis and drug design. For the next step of the research, collaborations with clinical teams are needed. A sufficient number of highly qualified clinical data are required for the correlation study.

It is the first paper, as far as we know, of the interpretation of HLA DNA by artificial intelligence to give a conceptual view of alleles.

## Electronic supplementary material

Below is the link to the electronic supplementary material.

Supplementary material 1 (DOCX 8 kb)

Supplementary material 2 (PPTX 501 kb)
