# Supplementary material for: Graphical classification of DNA sequences of HLA alleles by deep learning
Source: Hum Cell. 2018 Jan 11;31(2):102–5. doi: 10.1007/s13577-017-0194-6 (PMC5852191; doi:10.1007/s13577-017-0194-6)
Supplement: Supplementary file 1 — Supplementary material 1 (DOCX 8 kb) [file 13577_2017_194_MOESM1_ESM.docx]

**Supplemental figures and description**

<Figures>

Supplemental Figure 1. Reconstruction error

Supplemental Figure 2. Training error.

<Legend to figure>

Figure 1. Reconstruction error progresses in 2-fold cross validation. HLA-A genes are divided into two groups, A and B.

1a: numerical vector (training: group A, test: group B).

1b: numerical vector (training: group B, test group A).

1c: document vector (training: group A, test: group B).

1d: document vector (training: group B, test group A).

Figure 2. Training error progresses. All of the HLA-A genes are used for training.

2a: numerical vector.

2b: document vector.

Two types of stacked autoencoders were constructed because of the difference of input data lengths, 3288 of the numerical vector and 1024 of the document vector. To learn the numerical vectors, the stacked autoencoder consists of stages (1) 3288-2600-3288, (2) 1600-800-1600, (3) 800-400-800, (4) 400-150-400, (5) 150-2-150 autoencoders, corresponding to the stages in the figure. To learn the document vectors, the stacked autoencoder consists of (1) 1024-786-1024, (2) 786–384-786, (3) 384-128-384 and (4) 128-2-128 autoencoders, corresponding to the stages in the figure.

To determine the optimal number of training epochs, we divided 540 HLA-A genes into two groups of 270 genes and performed 2-fold cross validation. In 2-fold cross validation, the two groups are used for training and testing, respectively and alternately. With 80,000 epochs for the first trained autoencoder (1) for both of the stacked autoencoders and 30,000 epochs for the other autoencoders, both of the validation results show that the reconstruction error of testing data decreases and does not increase during training except for fluctuations but converges into a certain value as shown in Suppl. Fig. 1. The training epochs were empirically determined by several trials. The results mean that the given number of training epochs are adequate for our constructed neural network and the examined data, because overfitting did not occur. For final experiments (Fig. 2 and 3), all of the 540 genes are used for training and 0 for testing. Although testing cannot be done, as shown in Suppl. Fig. 2, the training error progress shows that the error drops very much in the early stage and converges in a certain value gradually. Therefore, it can be considered that 540 genes are learned well without overfitting.
