# Supplementary figures and images for: Graphical classification of DNA sequences of HLA alleles by deep learning
Source: Hum Cell. 2018 Jan 11;31(2):102–5. doi: 10.1007/s13577-017-0194-6 (PMC5852191; doi:10.1007/s13577-017-0194-6)

## Slide 1
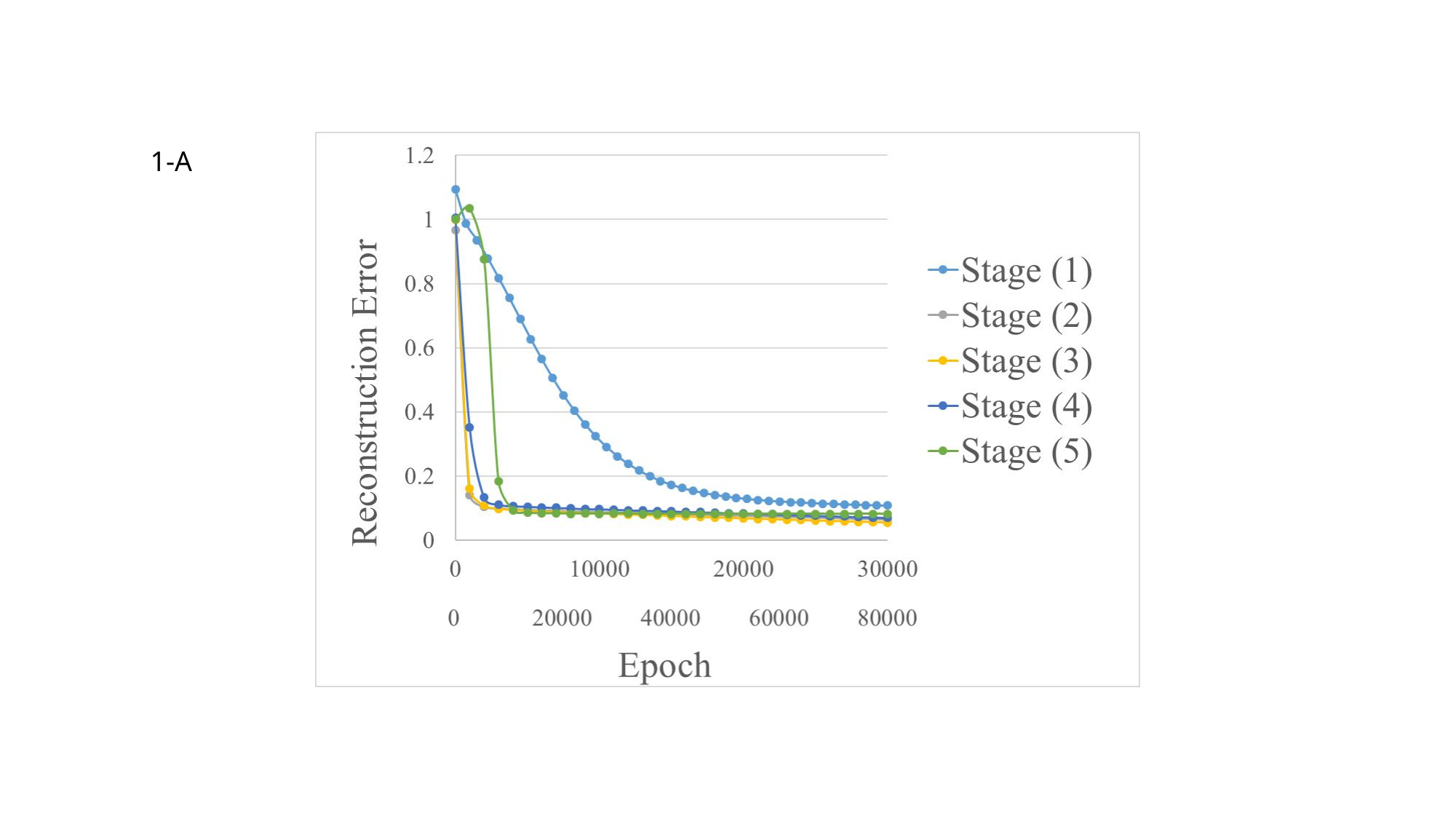

1-A

## Slide 2
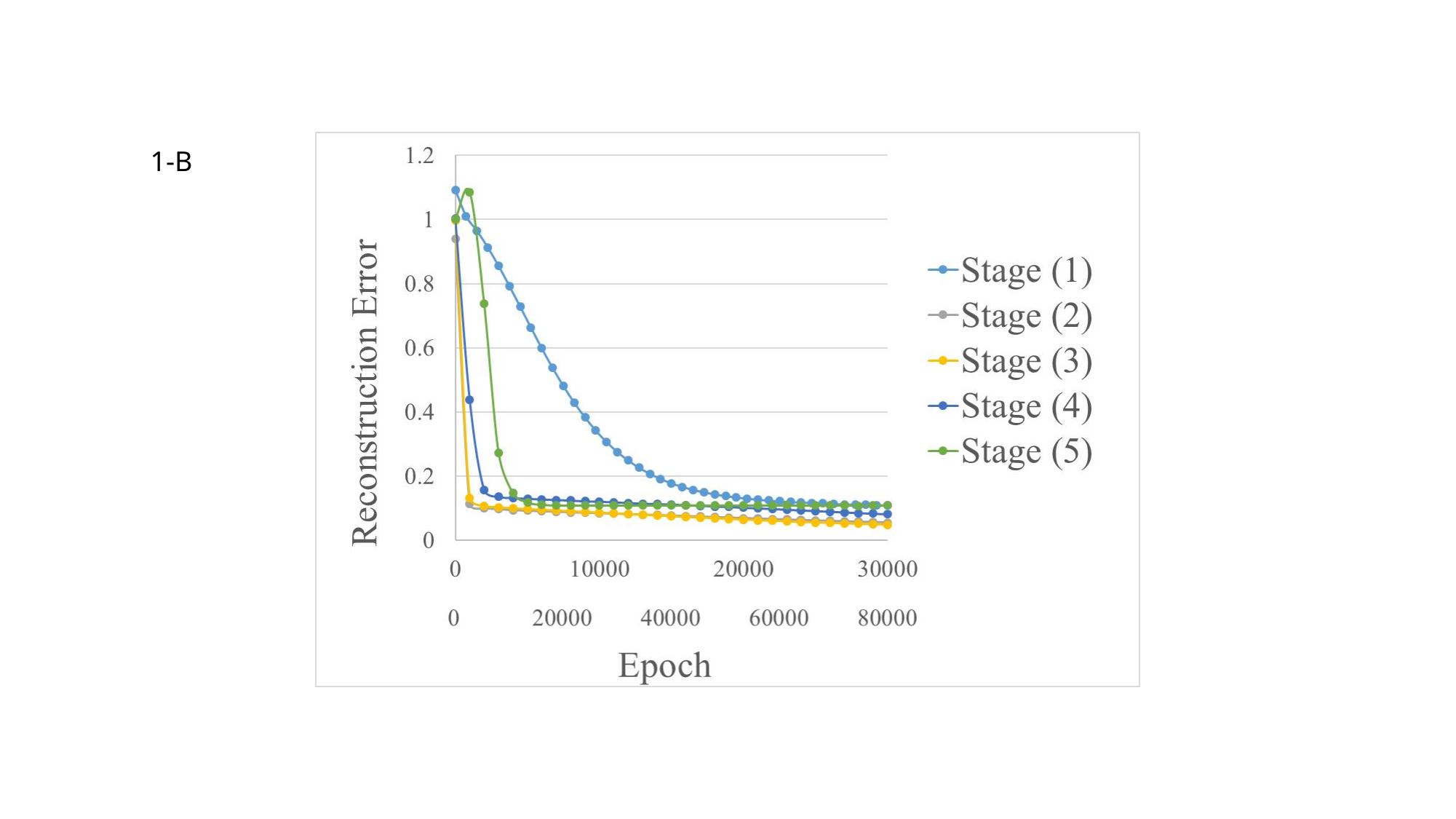

1-B

## Slide 3
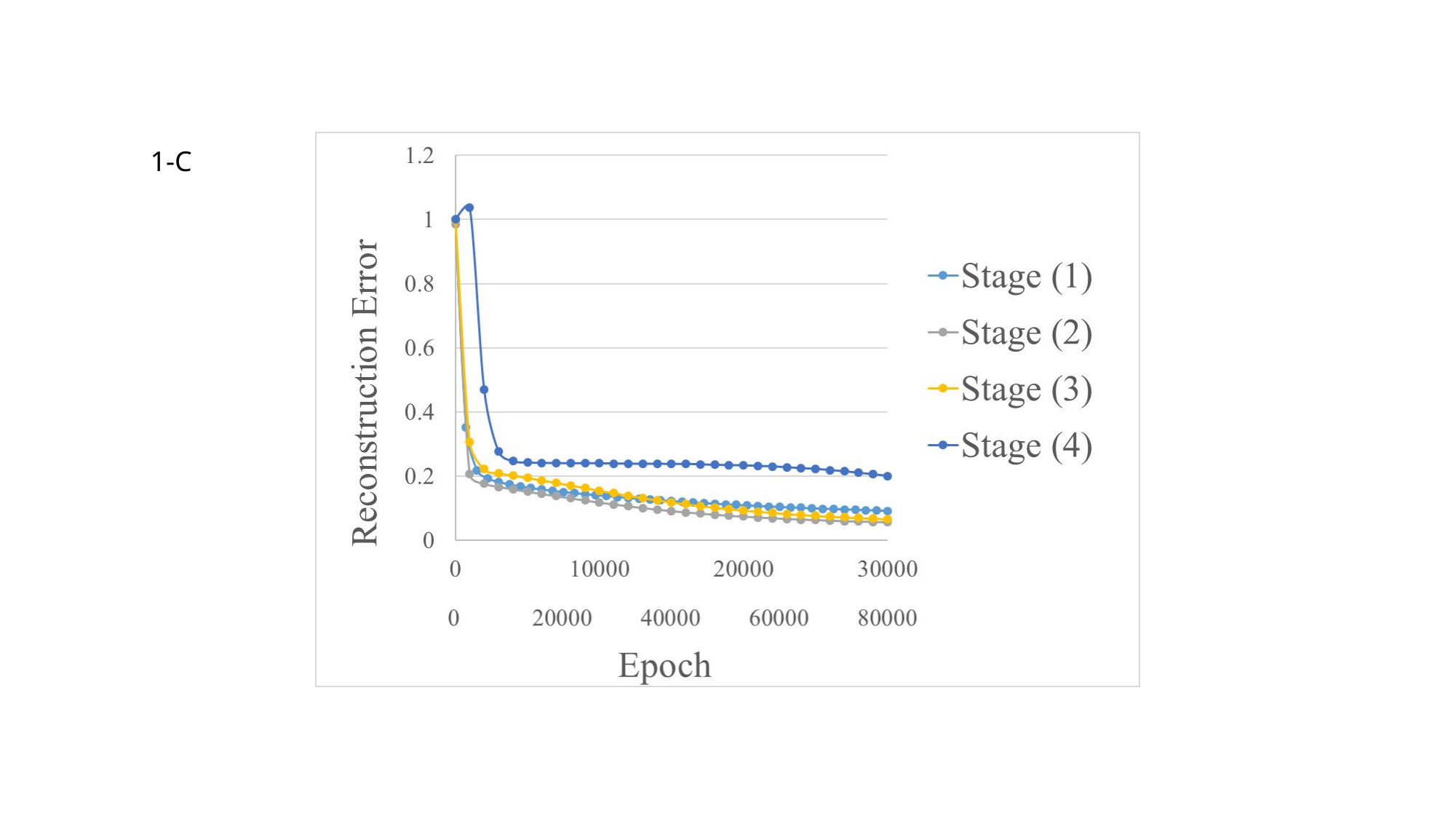

1-C

## Slide 4
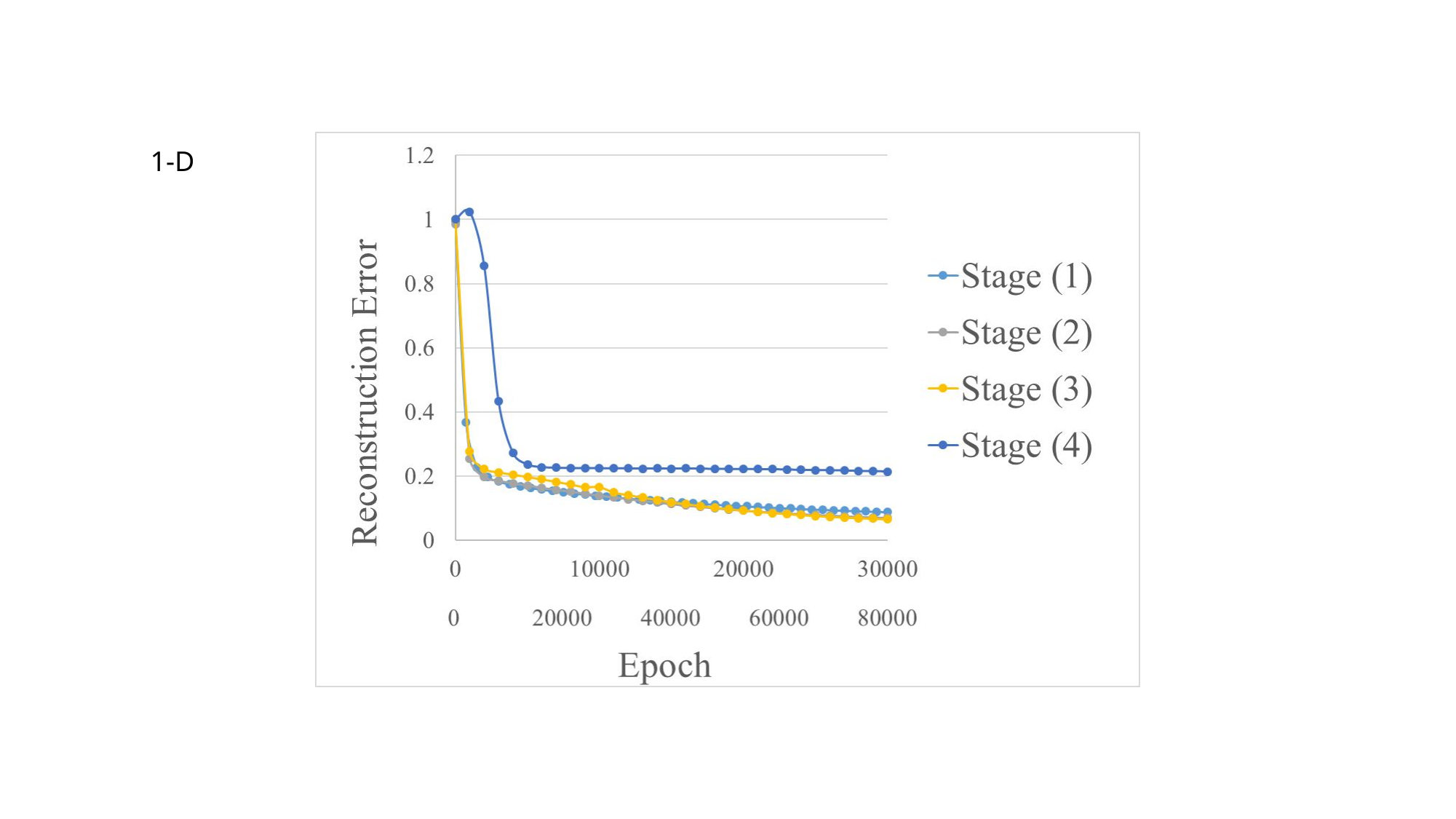

1-D

## Slide 5
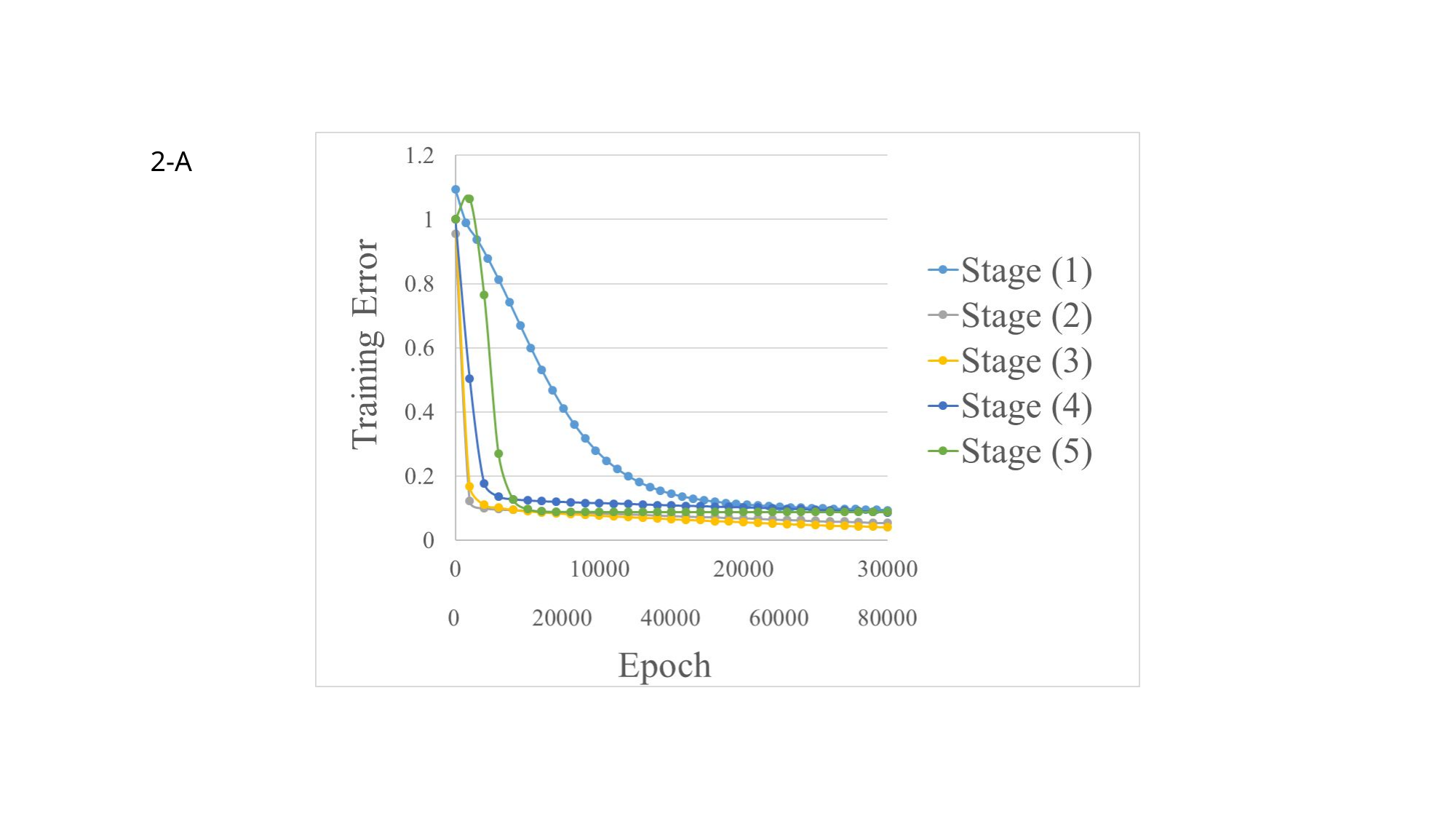

2-A

## Slide 6
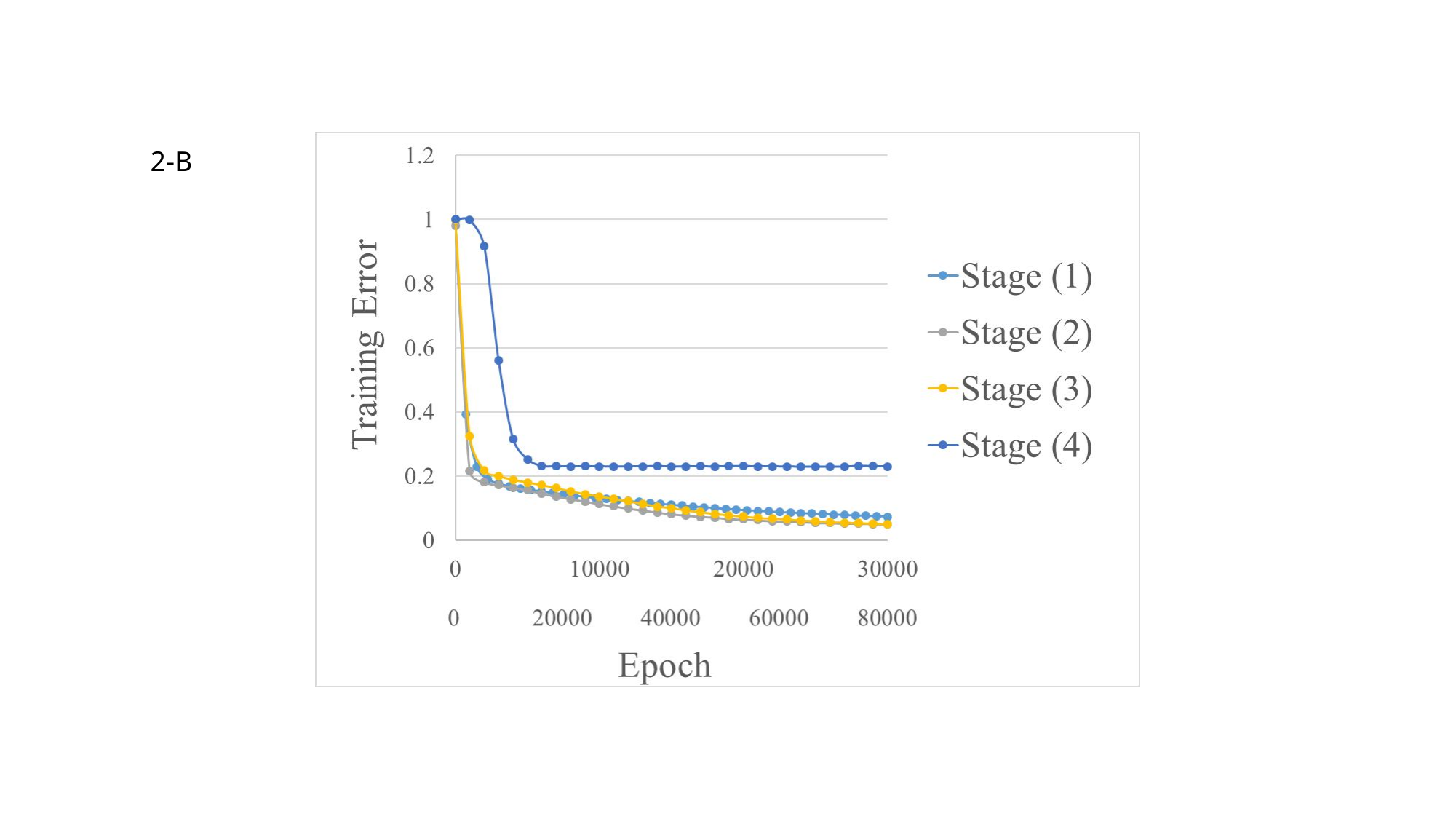

2-B

Supplement: Supplementary file 2 — Supplementary material 2 (PPTX 501 kb) [file 13577_2017_194_MOESM2_ESM.pptx]
